# Local Anesthetics

**Published:** 1899-10

**Authors:** J. S. Cassidy

**Affiliations:** Covington, Ky.


ARTICLE IX.

### LOCAL ANESTHETICS.

J. S. CASSIDY, D. D. S., COVINGTON, KY.

The introduction of local anesthetics to dental practice has probably not been an unmixed blessing. It has certainly enabled the unmitigated liar, with some show of truth, to add largely to his armament of deception, and has impressed the innocent patient, before the operation with the confiding belief that painless dentistry is an accomplished fact. It leads, therefore, to so-called quackery and to expectations on the part of the public that can only be partially fulfilled, and in some cases to possible damage suits for breach of contract.

With the incoming of cocain your essayist did his share in extolling the virtues of this much-abused drug, and at one time was considered by certain members of this society as little fietter than a horse thief, for stating that it was able to produce anesthetic efforts on dentin. After an experience with it, and by consultation with friends in regard to it, I cannot go back on the early position then taken, except to emphasize more fully the dangerous, as well as the beneficial qualities of this famous alkaloid.

The good things of this world, useful and beautiful, are all woefully abused by the wicked, ignorant and incompetent; whether we apply this aphorism to therapeutics, religion or politics, or the various aspects in which man is presented in his efforts to reach the objects of his unworthy desires, its truth is evident.

Local anesthesia is a good thing, provided it can be induced without loss of precious time and with comparative safety; but such approach to perfection in special therapeutics is not as yet attained, and probably never will be, and in consequence, those of us who practice in this direction should not only select the best means to reach success, but also be prepared, as far as possible, to counteract, by previous and subsequent treatment, any concomitant evil effects that may occur.

The present writer is personally not much interested in extracting teeth. It is proper to observe, however, that many of those engaged largely in that kind of practice prefer for injection, B. Eucain Hydrochlorid. It is less toxic than cocain preparations, and unlike the latter, causes local hyperæmia, supplying thus to the part an extra amount of defensive proteids, which probably prevent the immediate systemic poiaonous influence and excessive limited inflammation noticed so often as sequela to the injection of drugs which cause anæmia.

Inasmuch, however, as certain hydrochlorid is still used most freely in this way, might it not be good practice to administer some preantidote as a precaution against possible intoxication. Dr. Lennox Curtis says that by giving "volasem" to his patients before injecting cocain, all symptoms of bad effect from the latter are prevented. Volaseum is a propriety affair, the active principle of which is understood to be physostigma venenosum, the physiological antidote to belladonna, and as belladonna itself is one of the antidotes employed in cocain poisoning, the use of volasem in the way advised by Dr. Curtis might be regarded as in accordance with strictly scientific contrariety.

"Orthoform" is a new local anesthetic intended mainly for use in burns and other painful local affections. It may be applied safely and effectively in pyorrhea pockets, etc., before operating. We use it empirically, not knowing exactly what it is, except that it probably belongs to the benzene ring, by lateral chains, which, in therapeutics, means nothing much, for of the thousands of both natural and artificially manufactured organic compounds, all may be said to be derived from either methane ($CH_4$), or benzene ($C_6H_6$). Nevertheless, Dr. Prinz, in a late number of the "Ohio Journal," recommends orthoform the alkaloid, and orthoform hydrochlorid, the salt, in equal proportions dissolved in water, as a perfectly safe injection for painless removal of teeth.

It is hoped that in discussing this paper, the majority of this society will decide as to the value of "vapocain" in reducing sensibility of dentin. My own experience with it has not been altogether satisfactory. Theoretically, the ether should drive out the moisture from the tubuli and deposit the cocain salt therein, to be redissolved by the reappearing natural fluid of the tooth, and so secure effective anesthesia. Sometimes this theory agrees clinically in my office, but oftener otherwise, and I ask you to kindly tell your experience with it.

"Potossocain" is another preparation of cocain intended for a dentinal obtundant, which, although its alkalinity may be in its favor; is possessed also of the elements of uncertainty. Indeed, must we acknowledge that there has been really but slight improvement in this respect, aside from cataphoresis, over the early method of placing cocain hydrochlorid crystals in the tender cavity and allowing them to dissolve therein, under pressure? If this experience is yours, more than less, I shall go back to first principles until something better comes along.

It is high time that we arrive at some definite conclusion as to whether the medicines we work with, under given circumstances, are possessed of the power with which we are too often disposed to credit them, in either their physiological, chemical or dynamic influence.—*Indiana Dental Journal.*

